# The Prognostic Value of Histopathological Features in Early-Stage Mycosis Fungoides: Insights from a Retrospective–Prospective Cohort Study

**DOI:** 10.3390/dermatopathology11020017

**Published:** 2024-06-14

**Authors:** Sandra Jerkovic Gulin, Ivana Ilic, Romana Ceovic

**Affiliations:** 1 Department of Dermatology and Venereology, Ryhov County Hospital, 551 85 Jonkoping, Sweden; 2 Department of Biomedical and Clinical Sciences, Faculty of Medicine and Health Sciences, Linkoping University, 581 85 Linkoping, Sweden; 3 Department of Pathology and Cytology, University Hospital Centre Zagreb, 10000 Zagreb, Croatia; ricilic@gmail.com; 4 Department of Dermatology and Venereology, School of Medicine, University of Zagreb, University Hospital Centre Zagreb, 10000 Zagreb, Croatia; romana.ceovic@gmail.com

**Keywords:** mycosis fungoides, histopathological predictors, disease progression, skin lymphoma, prognostic factors

## Abstract

Primary cutaneous lymphomas (PCLs), especially mycosis fungoides (MF), pose significant diagnostic and therapeutic challenges. This study aims to correlate initial histological features with the disease course and survival in MF patients. A retrospective–prospective cohort study was conducted on 83 patients diagnosed with early-stage MF at the Departments of Dermatovenerology and Pathology, UHC Zagreb, from January 2003 to December 2012. The analyzed histopathological parameters included lichenoid dermal lymphocyte infiltrate, Pautrier microabscesses, and lymphocyte atypia. Patients with more than 30 guardian lymphocytes per 100 keratinocytes exhibited worse overall and progression-free survival. Furthermore, those with over 50% atypical lymphocytes demonstrated a faster progression rate. A dense lichenoid dermal infiltrate and a high count of lymphocyte “keepers” significantly increased the mortality risk within five years of diagnosis. This study did not fully confirm the hypothesis regarding the prognostic value of large Pautrier microabscesses but highlighted the importance of dense lichenoid infiltrates. The study identified new potential histopathological prognostic factors in early-stage MF, suggesting the need for larger studies to confirm these findings. The identification of such predictors could enhance the prognostic stratification and guide more tailored therapeutic approaches for MF patients.

## 1. Introduction

Primary cutaneous lymphomas (PCLs) represent extranodal neoplasms affecting T, NK, or B lymphocytes with a particular affinity for the skin. It is imperative to distinguish them from skin involvement in extracutaneous leukemias and lymphomas. PCLs, also referred to as skin lymphomas, encompass various types, and an accurate classification mandates the integration of several different features, especially correlating clinical manifestations and disease course with histopathological, immunohistochemical, and molecular data [1,2,3].

MF stands as the most prevalent T-cell skin lymphoma, constituting 50% of primary skin lymphomas [3]. MF encompasses various clinical stages, including premycotic, plaque-infiltrative, and tumor stages. Most patients with early-stage MF do not progress to advanced disease [4]. The incidence has shown regional variability and an increasing trend [5,6,7]. Despite numerous studies, the etiology of MF remains elusive, though associations with other hematological disorders and malignant and autoimmune diseases have been noted [8,9,10].

The diagnosis of MF relies on correlating clinical presentation, disease course, and histological and immunohistochemical analyses of skin biopsy samples. Notable histological criteria for diagnosing premycotic MF include Pautrier microabscesses, epidermal lymphocyte alignment, disproportionate epidermotropism, and lichenoid dermal infiltrates. However, diagnosing early-stage MF can be challenging due to similarities with inflammatory skin diseases [11].

MF is an epidermotropic T-cell skin lymphoma cytologically characterized by the proliferation of small to medium-sized pleomorphic, cerebriform lymphocytes. Several histological criteria for the diagnosis of early-stage MF include Pautrier microabscesses, lymphocytes aligned along the dermo-epidermal junction, intraepidermal lymphocytes larger than lymphocytes in the dermis, disproportionate epidermotropism (epidermotropic lymphocytes with scant spongiosis), intraepidermal lymphocytes with a clear area (halo lymphocytes) around the nucleus, enlarged papillary dermis with mild fibrosis and coarse collagen bundles, and a lichenoid dermal infiltrate of lymphocytes [12,13,14]. Intraepidermal clusters of lymphocytes (Pautrier microabscesses), which were considered a hallmark of the disease for decades, are present only in a smaller number of cases of premycotic-stage MF (prediagnostic stage) and often do not exist in advanced stages of the disease [15]. Early lesions of MF most often show a lichenoid infiltration of lymphocytes in the enlarged papillary dermis, hyperplastic, normal, or atrophic epidermis with a dominance of small lymphocytes. Atypical cells are extremely rare in early lesions. Also, solitary epidermotropism (epidermotropism of individual lymphocytes) is found significantly more often than Pautrier microabscesses in early lesions. Diagnostic criteria in the early stage of MF include (a) epidermotropic lymphocytes with nuclei that are larger than the nuclei of lymphocytes in the upper dermis; (b) lymphocytes arranged along the basal membrane of the epidermis; (c) intraepidermal lymphocytes in areas with scant spongiosis (disproportionate epidermotropism) [12,16]; (d) the expression of CD2, CD3, and CD5 in less than 50% of T lymphocytes; (e) the expression of CD7 in less than 10% of T lymphocytes; and (f) the aberrant expression of CD2, CD3, CD5, or CD7 on epidermal and dermal T lymphocytes [12]. Disproportionate epidermotropism can be seen in many inflammatory dermatoses. In about 5% of cases of early-stage MF, epidermotropism is minimal or non-existent, which may be a result of prior treatment. In the papillary dermis, pronounced fibrosis with coarse collagen fiber bundles, along with a band-like or patchy lichenoid infiltrate of lymphocytes, is found. Often, there is no edema of the dermis, eosinophils may be present but are not a common finding in patch and plaque lesions of early-stage MF, while the number of Langerhans cells is typically elevated [12,17].

MF plaques are characterized by the finding of a dense, band-like infiltrate of lymphocytes in the upper dermis, with a very frequent presence of Pautrier microabscesses and a dominance of small to medium-sized pleomorphic, cerebriform T lymphocytes [11,12,16].

In MF tumors, there is a dense, nodular, or diffuse infiltration of lymphocytes throughout the dermis, which commonly involves the subcutaneous fat tissue, while epidermotropism is often lost. Flat tumors are histopathologically characterized by the dense infiltration of lymphocytes only in the superficial and middle dermis, and in some cases, the infiltrate in the interstitium may dominate. In advanced stages of MF, patients may develop lesions containing numerous large cells, including immunoblasts, large pleomorphic cells, and large anaplastic cells [14,18,19,20,21]. The large-cell transformation of MF is defined by the presence of large cells making up more than 25% of the infiltrate or large cells forming microscopic nodes, and it is found in about 50% of cases of the tumor stages of MF [14,19,20,21,22,23]. The course of MF is indolent until large-cell transformation occurs, which is rare, and is associated with an aggressive clinical course and shortened survival [19].

MF is characterized by the infiltration of α/β helper memory T lymphocytes (βF1+, TSR-γ−, CD3+, CD4+, CD5+, CD8−, CD45Ro+, and TIA-1−). Rare cases express a T-cytotoxic (βF1−, TSR-γ−, CD3+, CD4−, CD5+, CD8+, and TIA-1+) or γ/δ (βF1−, TSR-γ+, CD3+, CD4−, CD5+, CD8+, and TIA-1+) phenotype, and neither clinically nor prognostically differ from the former [12,13,14,16,24,25,26]. The loss of pan-T-cell markers (CD2, CD3, and CD5) supports the diagnosis of MF and T-cell lymphomas but is very rare in early MF lesions [13,14,16,27]. Rarely, the early stage of MF shows an aberrant CD4+/CD8+ or CD4−/CD8− phenotype [16,28]. MF cases that are CD4−/CD8− may be positive for PD-1 [29,30]. Apart from MF, malignant lymphocytes in Sézary syndrome can also be PD-1-positive [29]. In the late stages of MF, lesions may contain numerous CD20+ B lymphocytes, which can even form germinal centers and mask the neoplastic T-lymphocyte infiltrate. Such cases must not be mistaken for B-cell skin lymphomas. Also, the occurrence of composite skin lymphomas, defined as neoplasms of T- and B-cell origin in one anatomical region or within the same lesion (e.g., the spectrum of T-cell neoplasms combined with different B-cell lymphomas/leukemias), should not be overlooked [13,14,16,28,31,32,33,34].

The immunohistochemical characteristics of MF cells are similar to those in many inflammatory skin diseases [11,35]. Staining with CD3 and CD4 markers will help highlight the epidermotropism of T lymphocytes, but intraepidermal lymphocytes cannot be considered pathognomonic for MF. It is believed that in the early stage of MF, there is a loss of CD7 antigen [36]. However, some studies have shown that inflammatory dermatoses can also exhibit the loss of CD7 antigen in some cases [11,37,38].

A recent study that investigated the possibility of distinguishing the premycotic stage MF (prediagnostic stage) from benign skin conditions based on histological criteria found that convoluted lymphocytes, subcutaneous infiltration, and follicular mucin are 100% specific for MF, and the absence of edema is 100% sensitive and a specific criterion for differentiating MF from inflammatory imitators [22,39].

A comprehensive study on early-stage mycosis fungoides revealed a common histopathological pattern of lichenoid dermal infiltrate with varied epidermotropism and highlighted the need for multiple biopsies and clinical–histological correlation to enhance diagnostic accuracy and support detailed immunohistochemical analysis [13]. It is advised to always perform more than one biopsy of different lesions to ensure enough material for further immunohistochemical and molecular procedures and to increase the sampling sites and diagnostic yield [12,13].

Numerous studies have failed to identify a reliable marker for the diagnosis of early-stage MF [40,41]. PCLs, particularly MF, present diagnostic and therapeutic challenges.

## 2. Objectives

The aim of this study is to systematically assess the prognostic implications of specific histopathological features observed in the initial biopsies of patients with early-stage MF (IA, IB, and IIA), including dense lichenoid dermal infiltrate of lymphocytes and the presence of Pautrier microabscesses (Darier’s nests). Through a detailed analysis combining retrospective and prospective cohort data, this research aims to establish a clear correlation between these early histological markers and the subsequent disease course and prognosis. By identifying these predictive indicators, this study endeavors to contribute to the enhancement of diagnostic accuracy, inform therapeutic strategies, and ultimately improve patient outcomes in early-stage MF.

## 3. Materials and Methods

In this retrospective–prospective cohort study, patients diagnosed with early-stage MF (stages IA, IB, and IIA) between January 2003 and December 2012 at the Clinic for Dermatovenerology and the Clinical Institute for Pathology and Cytology of the Clinical Hospital Center Zagreb were included. These patients had comprehensive clinical data and sufficient stored samples for additional histological and immunohistochemical analysis. This study comprised 83 patients. Excluded from the study were patients with a suspected diagnosis of MF that was not confirmed by histological analysis and clinical course, as well as those lacking adequate samples for further analyses.

The analysis of existing medical documentation included age, sex, clinical presentation, duration of the premycotic stage (prediagnostic stage MF), response to therapy, and occurrences of relapse and disease progression. The staging of the disease utilized the TNMB classification for MF/SS of the International Society for Cutaneous Lymphomas and the European Organisation for Research and Treatment of Cancer. Clinical presentation was defined by the type of skin lesions (patch, plaque, or tumor), the extent of skin lesions (≤10% of body surface area, >10% of body surface area), and lymph node size (enlarged, not enlarged). Disease progression was defined by at least one of the following criteria: (a) progression from plaque to tumor stage or erythroderma; (b) histologically confirmed lymph node involvement in patients previously limited to the skin; (c) visceral involvement in patients previously limited to the skin or lymph nodes; and (d) death due to lymphoma. Response to therapy was defined as (a) complete response (complete clinical regression of all MF lesions), (b) partial response (any response less than complete), or (c) no response (no visible clinical response to therapy).

Skin samples from patients stored in the archives of the Clinical Institute for Pathology and Cytology, Clinical Hospital Center Zagreb, were utilized. The samples were fixed in 10% buffered formalin, dehydrated in alcohol concentrations, embedded in paraffin blocks, and sliced into 4-micron-thick sections before being stained with the standard hematoxylin-and-eosin method, followed by immunohistochemical staining using commercially available antibodies for CD2 (clone AB75, Novocastra, UK), CD3 (clone F7.2.38, Dako, Denmark), CD4 (clone 4B12, Dako, Denmark), CD5 (clone 4C7, Dako, Denmark), CD7 (clone CBC.37, Dako, Denmark), and CD8 (clone C8/144B, Dako, Denmark). For antigen demasking, a microwave oven was used (95 °C, 15 min, target retrieval solution pH 9.0). Expression of markers was visualized using the standard avidin–biotin immunohistochemical method (LSAB, Dako, Glostrup, Denmark) with an automatic staining machine (TechMate, Dako) that utilizes capillary action. Positive reactions for antibodies CD2, CD3, CD4, CD5, CD7, and CD8 were identified by the membrane staining of tumor cells. Positive and negative control stains were performed, with lymph node tissue serving as the positive external control.

**Figure 1:**
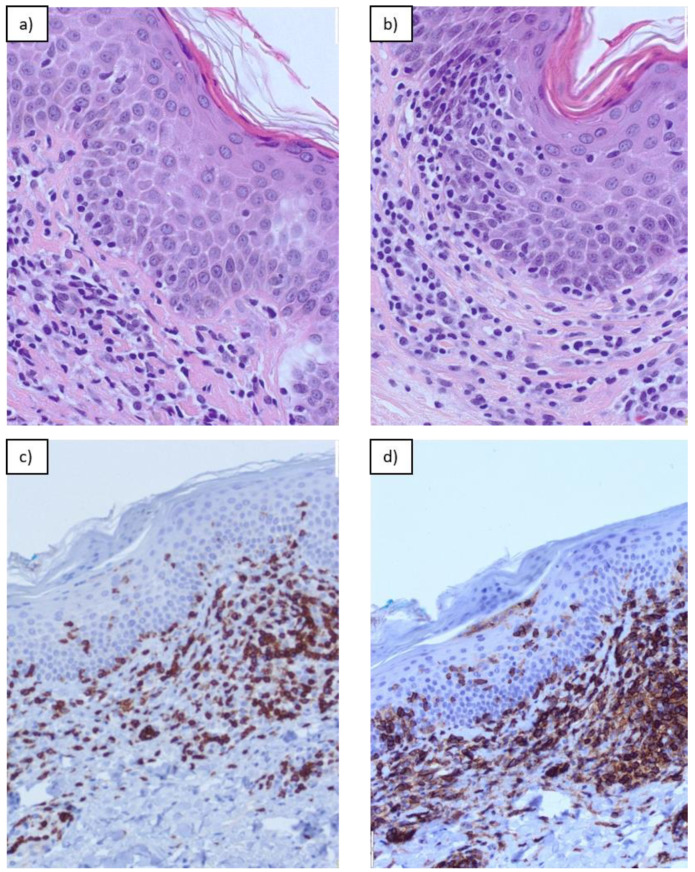
“Haloed” lymphocytes named lymphocyte “keepers” in the basal layer of the epidermis and lichenoid infiltrate in Mycosis fungoides: (**a**) H&E, magnification ×20; (**b**) H&E, magnification ×20; (**c**) cd3, magnification ×10; (**d**) cd4, magnification ×10.

In the examined preparations, the following histological and immunohistochemical parameters were analyzed: (1) the quantity of lichenoid dermal infiltrate of lymphocytes (classified semi-quantitatively as scant, moderate, or dense); (2) Pautrier microabscesses (intraepidermal collections of atypical lymphocytes) classified as absent or the presence of small (3–10 atypical lymphocytes per cluster) or large (more than 10 atypical lymphocytes) lymphocytes; (3) “haloed” lymphocytes named lymphocyte “keepers” in the basal layer of the epidermis (at least 4 lymphocytes in a row) presented as the number of consecutive lymphocytes per number of keratinocytes (Figure 1); and (4) the amount of lymphocyte atypia determined semi-quantitatively as no lymphocyte atypia, ≤10%, ≤50%, or >50% of lymphocytes showing signs of atypia. These histological criteria were quantified in the preparations across 10 visual fields.

For statistical analysis, appropriate parametric and non-parametric tests were used. Results with *p*-values less than 0.05 (*p* < 0.05) were considered statistically significant. The impact of each observed variable on disease progression and mortality was analyzed. Qualitative variables are presented in absolute numbers and percentages. Their association with progression and mortality was tested using the chi-square test. Quantitative variables are presented as the median and corresponding range. Their differences regarding progression and mortality were tested using the non-parametric Mann–Whitney U test. The correlation between the initial stage of the disease and the stage after progression was examined using the non-parametric Spearman correlation. The relative risk for the examined outcome (progression or death) for each variable was analyzed using logistic regression. Survival and time to progression were determined using Kaplan–Meier survival curves, and the differences in survival between groups of patients were examined using the log-rank test and chi-square test. Statistical data analysis was performed on a personal computer using Statistica for Windows, ver. 6.0.

## 4. Results

**Figure 2:**
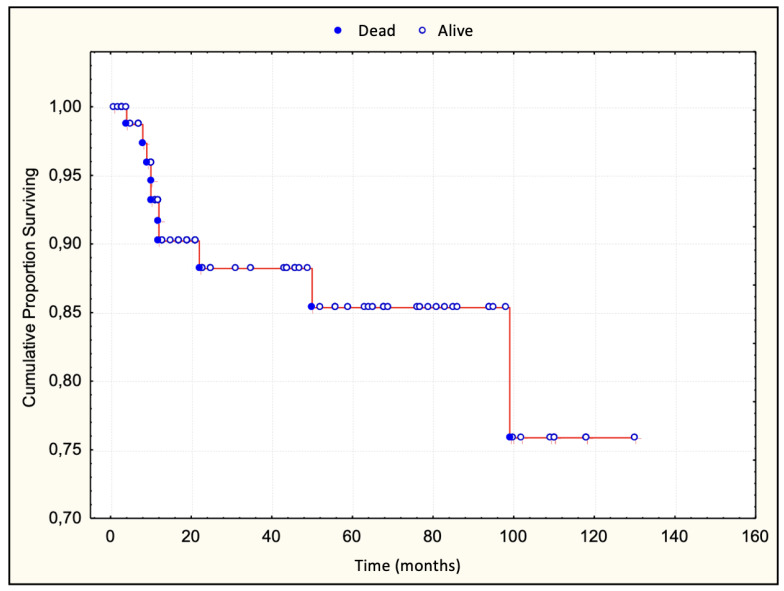
Overall survival curve.

This study included 83 patients aged between 7 and 85 years, with an average age of 60 years (Table 1). Of these, 49 were males, with an average age of 63 years (7–85), and 34 were females, with an average age of 59.5 years (25–82). At the time of diagnosis, 27 patients were in stage IA, 35 patients in stage IB, and 21 patients in stage IIA. The median follow-up of patients was 25 months (range 1–130). In 36 (43%) patients, the disease progressed. The median survival without disease progression was 48 months. Ten patients (12%) died, all from lymphoma following disease progression. The five-year survival rate was 86% (Figure 2). The median survival after progression was 58 months.

## 5. Survival

**Figure 3:**
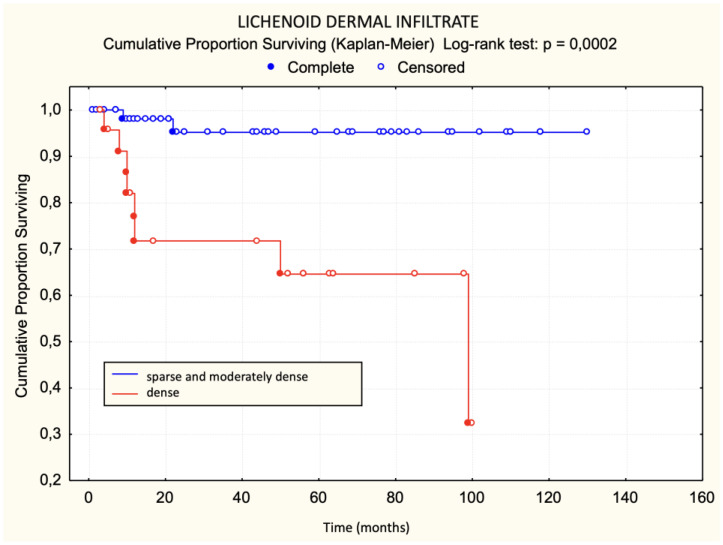
Lichenoid dermal infiltrate—survival.

**Figure 4:**
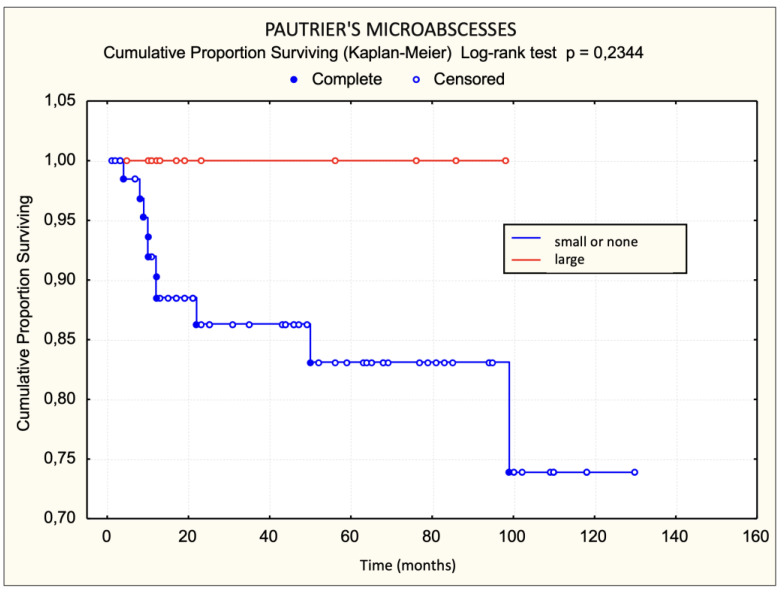
Pautrier microabscesses—survival.

**Figure 5:**
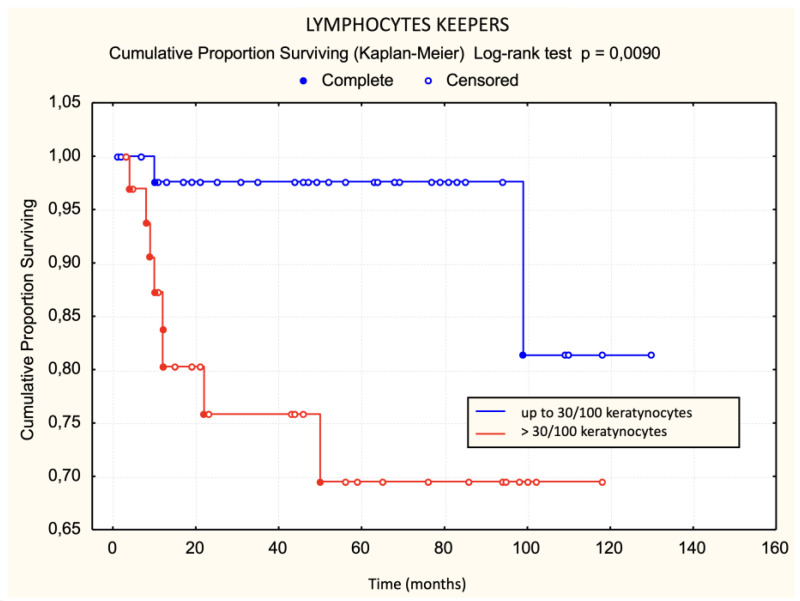
Lymphocyte “keepers”—survival.

Patients with a dense lichenoid dermal infiltrate had worse survival than those with sparse and moderately dense lichenoid dermal infiltrate. The difference was statistically significant (Figure 3). None of the patients with large Pautrier microabscesses died during the observed period (Figure 4). Patients with more than 30 lymphocyte “keepers” per 100 keratinocytes had worse survival than those with 30 or fewer lymphocyte “keepers” per 100 keratinocytes. The difference was statistically significant (Figure 5). Patients with more than 50% atypical lymphocytes exhibited a trend toward worse survival than those with 50% or fewer atypical lymphocytes, but the difference was not statistically significant.

Using the log-rank test, statistically significant unfavorable prognostic factors for survival were disease stage IIa, plaques in the initial clinical picture, a premycotic phase shorter than 4 years, enlarged lymph nodes, dense lichenoid dermal infiltrate, a large number of halo lymphocytes, a lack of response to treatment, and the occurrence of disease progression. Of these, the lack of response to treatment was the most significant, followed by pretherapeutic factors like dense lichenoid dermal infiltrate (Table 2).

If the data were analyzed using univariate logistic regression, both parameters listed in Table 3 increased the risk of mortality within the first five years after diagnosis.

## 6. Progression

**Figure 6:**
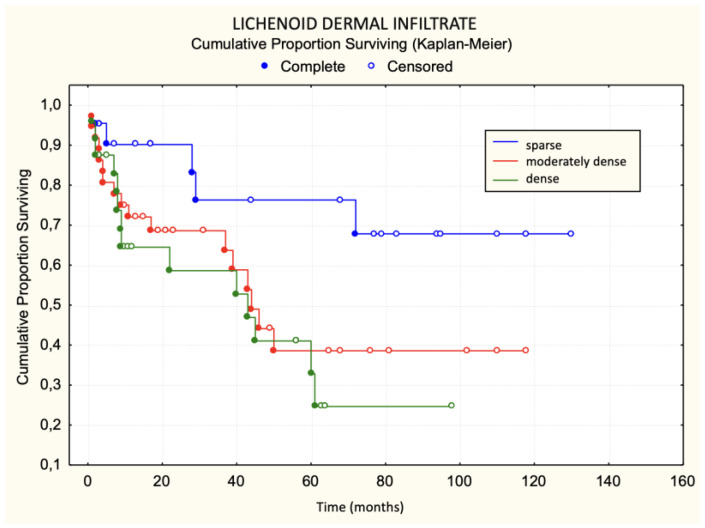
Lichenoid dermal infiltrate—progression.

**Figure 7:**
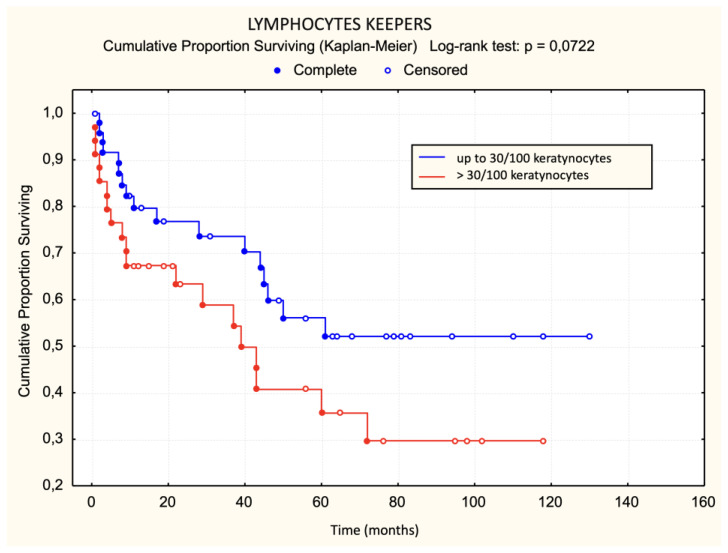
Lymphocyte “keepers”—progression.

**Figure 8:**
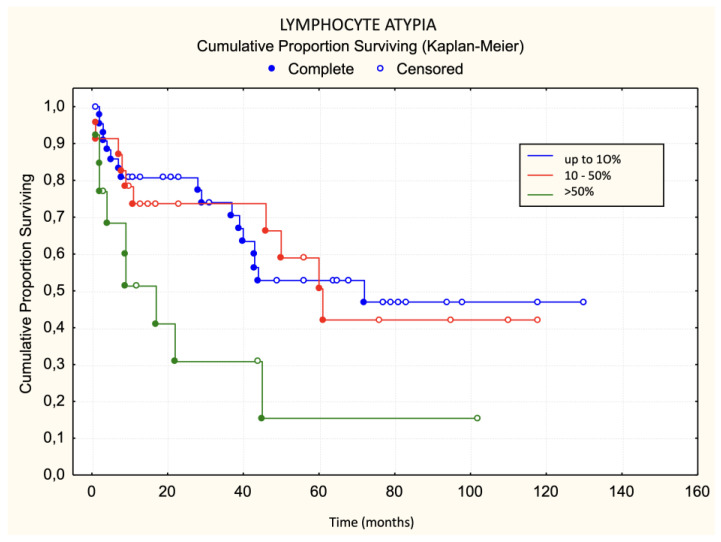
Lymphocyte atypia—progression.

**Figure 9:**
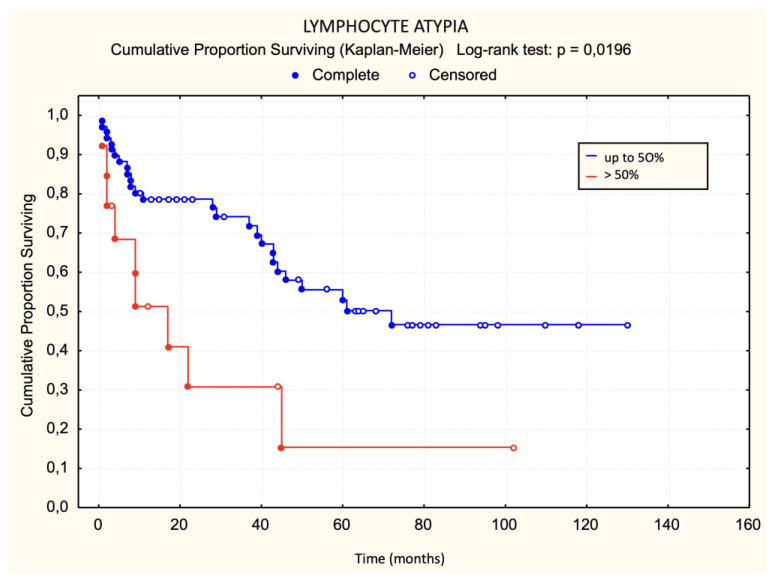
Lymphocyte atypia—progression.

There was no statistically significant difference in the occurrence of disease progression in patients divided into three groups based on the characteristics of the lichenoid dermal infiltrate (chi-square test: *p* = 0.0881, Figure 6). There was no statistically significant difference in the occurrence of disease progression in patients divided into two groups based on the characteristics of the lichenoid dermal infiltrate. There was no statistically significant difference in disease progression regarding Pautrier microabscesses when patients were divided into three groups (chi-square: *p* = 0.4708). There was no statistically significant difference in progression regarding Pautrier microabscesses even when patients were divided into two groups. Patients with more than 30 lymphocyte “keepers” per 100 keratinocytes exhibited a trend of faster progression than those with 30 or fewer lymphocyte “keepers” per 100 keratinocytes. The difference was on the border of statistical significance (Figure 7). The difference in progression-free survival between three groups of patients divided according to the proportion of atypical lymphocytes was on the border of statistical significance (chi-square: *p* = 0.0513, Figure 8). Patients with more than 50% atypical lymphocytes had faster progression than those with 50% or fewer atypical lymphocytes. The difference was statistically significant (Figure 9).

Using the log-rank test, statistically significant unfavorable prognostic factors for progression-free survival was a large number of atypical lymphocytes (Table 4). None of the variables examined listed in Table 5 were significant for the risk of disease progression.

## 7. Discussion

In our single-center cohort retrospective–prospective study, we assessed 83 patients over a median period of nearly 2 years. The study’s limitations include its single-center design, relatively brief follow-up duration, modest cohort size, and a small number of deceased subjects (*n* = 10), which constrained the logistic regression analysis for mortality. However, a strength of our research lies in the comprehensive data availability for all investigated variables across participants, enabling complete analyses for the cohort (*N* = 83). Despite its mixed retrospective–prospective nature, accurate records of disease progression or mortality were obtained for all cases, facilitating the precise application of the Kaplan–Meier survival analysis. We observed disease progression in 43% of the cohort (*n* = 36), with a median post-progression survival of 58 months, and a five-year survival rate of 86%. Consistent with prior findings, early-stage MF (IA-IIA) typically predicts favorable outcomes with survival spanning 10–35 years, though approximately 25% of cases escalate to advanced stages, resulting in a median survival under 4 years. Subjects with lymph node involvement exhibited a median survival of merely 13 months, aligning with established prognoses indicating worse outcomes for patients with advanced disease stages, higher TNMB classifications, extensive skin involvement, or appearance of changes [2,4,14,20,21,25,42,43,44,45,46,47]. In studies on MF and Sézary syndrome, the key prognostic factors for disease progression were identified. Patient age, TNMB classification, and the presence of extracutaneous disease were significant predictors [9]. Agar et al. highlighted survival differences based on lesion types in early-stage MF [47].

In our study, we attempted to determine histological criteria that influence survival and disease progression. Patients with dense lichenoid infiltrates more frequently had fatal outcomes compared to patients with moderately dense and sparse lichenoid dermal infiltrates. None of the patients with sparse lichenoid dermal infiltrates died. Contrary to our expectations, there was no statistically significant difference in disease progression based on sparse, moderately dense, and dense lichenoid dermal infiltrate. Previous studies have not investigated the impact of lymphocytic lichenoid dermal infiltrates on survival and disease progression, making these results exceptionally valuable.

Contrary to expectations, we did not find a statistically significant difference in survival and disease progression based on the presence of small and large Pautrier microabscesses. None of the patients with large Pautrier microabscesses died during the observed period. Vonderheid et al. conducted a study on 33 patients with early-stage MF exhibiting disease progression, comparing them to 70 control patients without progression. They delineated negative prognostic indicators as large Pautrier microabscesses, atypical lymphocytes with distinct nuclear features in the dermal infiltrate, a dermal infiltrate composition of less than 20% CD8+ cells, and serum IgE levels exceeding 122 U/mL [17,19,48,49]. Our results only partially confirm the results of that study. Our study found that patients with more than 50% atypical lymphocytes had faster progression than those with 50% or fewer atypical lymphocytes, but it did not identify large Pautrier microabscesses as a negative prognostic factor associated with faster disease progression.

The analysis of lymphocyte “keepers” count showed the value of this histological criterion. Patients with more than 30 lymphocyte “keepers” per 100 keratinocytes had poorer survival and faster disease progression than those with 30 or fewer lymphocyte “keepers” per 100 keratinocytes. The impact of this criterion on survival and disease progression has not been analyzed in previous studies, making these results exceptionally valuable.

Patients with more than 50% atypical lymphocytes in the first biopsy had faster progression than those with 50% or fewer atypical lymphocytes. Although the results of comparing progression among the three groups of patients (<10%, 10–50%, and >50%) were not statistically significant, it should be noted that our sample size had a small number of deceased patients (*n* = 10). These results are consistent with previous research [17].

The results of univariate logistic regression for the basic model of mortality outcome within five years of diagnosis for MF show that dense lichenoid dermal infiltrate and >30 lymphocyte “keepers” per 100 keratinocytes increase the risk of mortality within the first five years after diagnosis.

The results of univariate logistic regression for the basic model of disease progression within 5 years of diagnosis for MF show that the presence and number of lymphocyte “keepers” greater than 30 per 100 keratinocytes, as well as atypical lymphocytes >50%, increase the risk of disease progression within 5 years of diagnosis, while large Pautrier microabscesses reduce this risk. Although these results were at the threshold of statistical significance, they indicated an association with progression.

This study has fulfilled the specified specific objectives. We determined which morphological features in the initial biopsy represent unfavorable prognostic factors for survival (dense lichenoid dermal infiltrate, a large number of lymphocyte “keepers”) and survival without progression (a large number of atypical lymphocytes).

The hypothesis that patients with early-stage MF who have a dense lichenoid dermal infiltrate of lymphocytes and large Pautrier microabscesses in the initial biopsy will have a more aggressive course of the disease and a poorer prognosis was not fully confirmed. Patients with dense LDI had a poorer prognosis, but none of those with large PM (*n* = 12) died. Regarding disease progression, the thesis that progression occurred more frequently and rapidly in those with dense or moderately dense LDI than in patients with sparse LDI was proven, while large PM was not associated with disease progression.

## 8. Conclusions

This study suggests new prognostic factors for the early stage of MF. Larger studies are needed to confirm and implement these data into histological criteria that would become standard in diagnosis to identify patients at increased risk of disease progression and poorer prognosis. The identification of these prognostic factors may also serve to create a better prognostic index for MF.

## Figures and Tables

**Table 1:** Patient characteristics and list of histological and immunohistochemical parameters analyzed in the study.

Variable	Patients (*N* = 83)
Age (years)	60 (7–85)
Gender	
Male	49 (59%)
Female	34 (41%)
Disease Stage	
Ia	27 (33%)
Ib	35 (42%)
IIa	21 (25%)
Clinical Presentation	
Patch	27 (33%)
Plaque	56 (67%)
Tumor	0
Duration of Premycotic Stage (Prediagnostic Stage) (months)	48 (0–240)
Extent of Lesions	
≤10% of skin surface	26 (31%)
>10% of skin surface	57 (69%)
Enlarged Lymph Nodes	21 (25%)
Disease Progression	36 (43%)
Fatal Outcome	10 (12%)
Therapeutic Response	
No response	9 (11%)
Partial response	37 (45%)
Complete response	37 (45%)
Lychenoid Dermal Infiltrate	
Sparse	22 (26%)
Moderately dense	37 (45%)
Dense	24 (29%)
Pautrier Microabscesses	
None	33 (40%)
Small	38 (46%)
Large	12 (14%)
Lymphocyte “Keepers” (Present)	
≤30/100 keratinocytes	49 (59%)
>30/100 keratinocytes	34 (41%)
Lymphocyte Atypia	
None	3 (4%)
≤10%	44 (53%)
10–50%	23 (28%)
>50%	13 (16%)

**Table 2:** Differences in survival based on all examined variables.

	Survived *n* = 73	Died *n* = 10	Log-Rank Test (*p*-Value)	
Lichenoid Dermal Infiltrate				
Sparse	22	0	***p* = 0.0484**	
Moderate	35	2		
Dense	16	8		***p* = 0.0002**
Pautrier Microabscesses				
None	28	5	*p* = 0.3182	
Small	33	5		
Large	12	0		0.2344
Lymphocyte “Keepers” (present)	55	8	*p* = 0.8491	
≤30/100 keratinocytes	47	2	***p* = 0.0090**	
>30/100 keratinocytes	26	8		
Lymphocyte Atypia				
None	3	0		
≤10%	40	4	Chi-square: *p* = 0.1469	
10–50%	20	3		
>50%	10	3		*p* = 0.1524

Bold *p*-Value: *p* < 0.1.

**Table 3:** Univariate logistic regression for the basic model of mortality outcome within 5 years in patients with MF.

Variable	RR	95% CI Lower	95% CI Upper	*p*-Value
Lichenoid dermal infiltrate—dense	11.77	2.05	67.61	0.0065
Lymphocyte “keepers” >30/100 keratinocytes	14.59	1.60	132.99	0.0184

**Table 4:** Differences in the occurrence of disease progression based on all examined variables.

	Progression *n* = 36	No Progression *n* = 47	Log-Rank Test *p*-Value	
Lichenoid Dermal Infiltrate				
Sparse	5	17	Chi-square *p* = 0.0881	*p* = 0.1146
Moderate	17	20		
Dense	14	10		
Pautrier Microabscesses				
None	14	19	Chi-square *p* = 0.4708	
Small	19	19		
Large	3	9		*p* = 0.2664
Lymphocyte “Keepers“ (present)	30	33	*p* = 0.1438	
≤30/100 keratinocytes	17	32	*p* = 0.0722	
>30/100 keratinocytes	19	15		
Lymphocyte atypia				
Absent	0	3		
≤10%	17	27	Chi-square *p* = 0.0513	
10–50%	10	13		
>50%	9	4		***p* = 0.0196**

Bold *p*-Value: *p* < 0.1.

**Table 5:** Univariate logistic regression for the basic model of disease progression within 5 years in MF.

	Relative Risk (RR)	95% Confidence Interval	*p*-Value
Pautrier microabscesses—large	0.222	0.040–1.228	0.0834
Lymphocyte “keepers”—present	4.105	0.947–17.794	0.0588
Lymphocyte “keepers” >30/100 keratinocytes	2.598	0.862–7.833	0.0885
Lymphocyte atypia >50%	3.750	0.866–16.230	0.0761

## Data Availability

Data supporting reported results are available upon request from authors.
